# Drug repositioning of mesalamine via supramolecular nanoassembly for the treatment of drug-induced acute liver failure

**DOI:** 10.7150/thno.101358

**Published:** 2025-01-01

**Authors:** Byeongmin Park, Eun Hye Kim, Hochung Jang, Yelee Kim, Youngri Ryu, Jiwoong Choi, Dongwon Shin, Myung Chul Lee, Yoosoo Yang, Kwangmeyung Kim, Sangmin Lee, Sun Hwa Kim, Man Kyu Shim

**Affiliations:** 1 KU-KIST Graduate School of Converging Science and Technology, Korea University, Seoul 02841, Republic of Korea.; 2 Medicinal Materials Research Center, Biomedical Research Division, Korea Institute of Science and Technology (KIST), Seoul 02792, Republic of Korea.; 3 Department of Life Sciences, Korea University, Seoul 02841, Republic of Korea.; 4 Division of Bio-Medical Science and Technology, KIST School, Korea University of Science and Technology, Seoul 02792, Republic of Korea.; 5 College of Pharmacy, Graduate School of Pharmaceutical Sciences, Ewha Womans University, Seoul 03760, Republic of Korea.; 6 College of Pharmacy, Graduate School of Pharmaceutical Sciences, Kyung Hee University, Seoul 02453, Republic of Korea.

**Keywords:** Acute liver injury, Supramolecular assembly, Prodrug, Mesalamine, Drug repositioning

## Abstract

**Rationale:** Acute liver failure (ALF) is characterized by rapid hepatic dysfunction, primarily caused by drug-induced hepatotoxicity. Due to the lack of satisfactory treatment options, ALF remains a fatal clinical disease, representing a grand challenge in global health.

**Methods:** For the drug repositioning to ALF of mesalamine, which is clinically approved for the treatment of inflammatory bowel disease (IBD), we propose a supramolecular prodrug nanoassembly (SPNs). Mesalamine is modified with a functional peptide of the FRRG sequence. The resulting mesalamine prodrugs form nanoassemblies solely through intermolecular interactions, ensuring high drug loading capacity and reducing the potential toxicity associated with the carrier materials of conventional nanoparticle systems.

**Results:** In acetaminophen (APAP)-induced ALF mouse models, the SPNs predominantly accumulate in injured target tissues owing to the nanoparticles' propensity to target the liver. Subsequently, cathepsin B overexpressed in hepatocytes by drug-induced inflammation triggers the release of mesalamine from the nanoassemblies *via* enzymatic cleavage, resulting in remarkable therapeutic efficacy. Meanwhile, nonspecific drug release in healthy cells is inhibited due to their relatively lower cathepsin B expression, which helps prevent the exacerbation of the ALF by minimizing adverse events related to drug exposure.

**Conclusions:** This study provides valuable insights into designing rational nanomedicine for repurposing mesalamine in ALF treatment, potentially inspiring further research to discover effective and safe therapeutic options for patients.

## Introduction

Acute liver failure (ALF) is the clinical manifestation of severe and sudden hepatic injury arising from various causes 1. A leading cause of ALF is an adverse reaction to medications, which can occur either predictably (non-idiosyncratic) when individuals are exposed to toxic doses of certain compounds or unpredictably (idiosyncratic) with many commonly used drugs 2. With the recent aging of the population and the resulting increase in exposure to many medications, the incidence of non-idiosyncratic ALF has significantly risen 3. The main example is acetaminophen (APAP) hepatotoxicity, which accounts for approximately 50% of ALF cases in Western countries 4. There are no satisfactory treatment options available for curing ALF other than liver transplantation, leaving it a fatal clinical disease 5.

APAP hepatotoxicity following an overdose involves centrilobular hepatocytes, where cytochrome P450 family 2 subfamily E member 1 (Cyp2E1) generates the reactive metabolite N-acetylbenzoquinoneimine (NAPQI), leading to glutathione depletion and the overwhelming accumulation of NAPQI adducts 6. These molecular changes induce the directional release of superoxide, which activates the mitogen-activated protein kinase (MAPK) cascade, culminating in mitochondrial membrane depolarization due to the activation and translocation of c-Jun N-terminal kinase (JNK) 7. Persistent JNK activation and translocation promote the mitochondrial permeability transition, and the subsequent release of endonuclease G and apoptosis-inducing factor (AIF) to the nucleus causes DNA fragmentation and ultimately cell death 8. Therefore, extensive studies have focused on developing promising drugs to regulate MAPK, which plays a central role in the progression of ALF, to provide treatment options for effectively counteracting the refractory disease currently faced in clinical settings 9-11.

Identifying additional medicinal indications of existing drugs can offer a better risk-versus-reward trade-off compared to developing new medicines 12. Mesalamine, a clinically approved drug for inflammatory bowel disease (IBD) treatment, inhibits the MAPK pathways, particularly targeting key mediators of JNKs 13. Additional beneficial effects of mesalamine in restoring disrupted epithelial layers in inflamed colons by effectively regulating inflammatory responses have been reported 14. In this context, we sought to repurpose mesalamine as a potential treatment for mitigating APAP-induced hepatotoxicity and thereby treating ALF. However, mesalamine may have limited efficacy due to unfavorable *in vivo* pharmacokinetics of small molecule structure, and nonspecific drug exposure could increase the risk of exacerbating ALF.

**Scheme 1:**
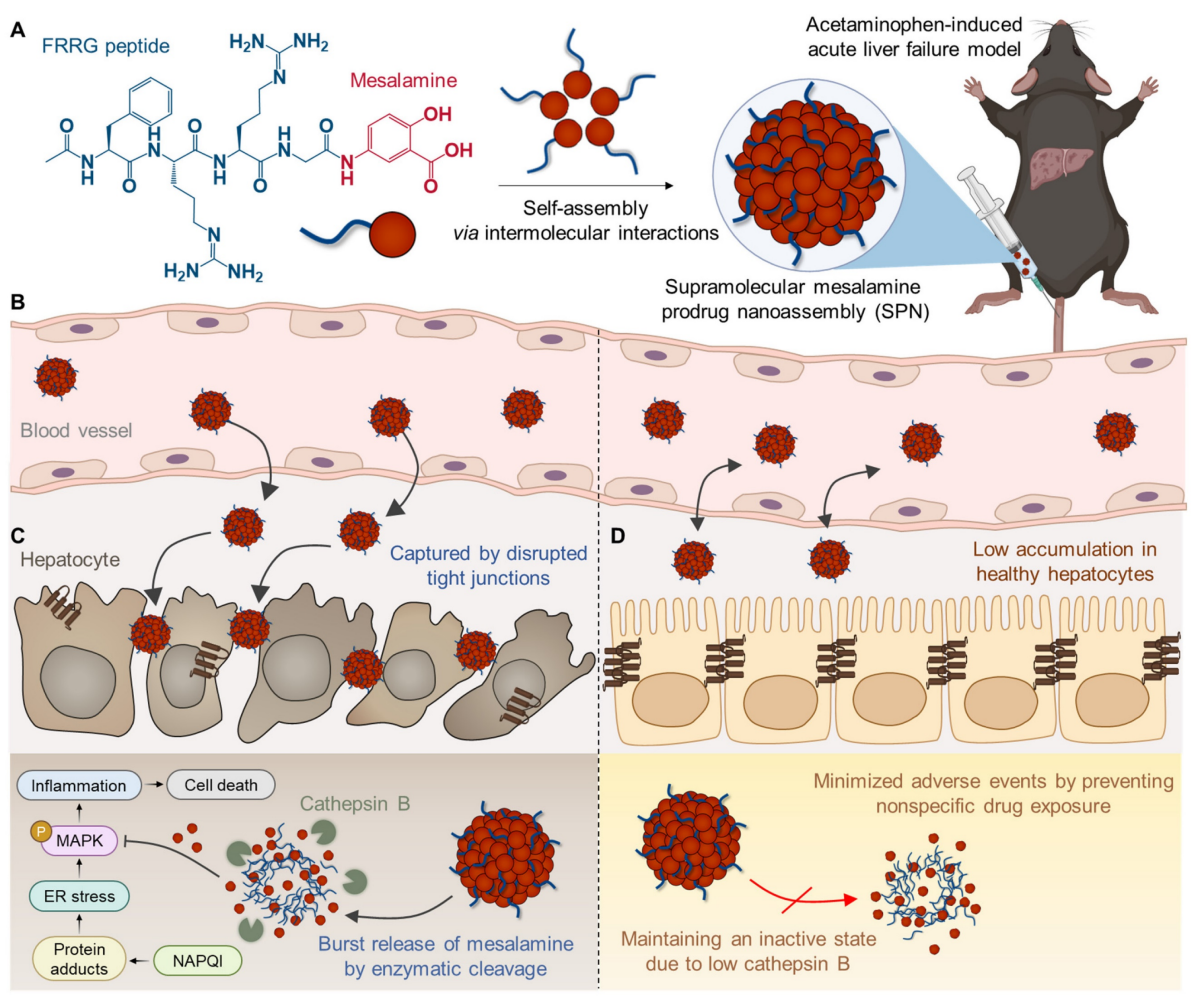
**Structure and action mechanism of SPNs for the treatment of ALF. (A)** Chemical structure of the mesalamine prodrug composing SPNs. **(B-D)** Processes involved in SPN-mediated alleviation of APAP-induced ALF. **(B)** The SPNs significantly accumulate in liver tissues due to extravasation and are further captured by the interstices between disrupted tight junctions during the onset of ALF. **(C)** Cathepsin B overexpressed in hepatocytes injured by APAP triggers the burst release of mesalamine through enzymatic cleavage of the prodrug. **(D)** Meanwhile, SPNs remain in an inactive state in healthy hepatocytes due to their relatively low cathepsin B expression, thereby minimizing adverse events from nonspecific drug exposure.

Herein, we propose a supramolecular mesalamine prodrug nanoassembly (SPNs) for ALF treatment. Mesalamine is modified with a functional peptide consisting of i) F (*Phe*) to promote intermolecular π-π interactions for the self-assembly of the system 15, ii) RR (*Arg-Arg*) as a substrate peptide for cathepsin B (Cat-B) 16 and iii) G (*Gly*) to enhance target enzyme sensitivity 15 **(Scheme 1A)**. The resulting conjugates spontaneously self-assemble into nanoparticles, ensuring high drug-loading content and minimizing adverse events by excessive carrier materials used in conventional nanoparticle systems, such as polymers, lipids and various organic/inorganic substances 17. From an industrial perspective, there is a significant advantage in overcoming the fundamental limitations of existing nanoparticles, which have been restricted in clinical translation owing to challenges in optimizing processes for quality control (QC) and scalable production 18.

Following intravenous injection in the APAP-induced ALF mouse model, SPNs accumulate within the inflamed tissues along to nanoparticles' propensity to target the liver through extravasation **(Scheme 1B)** 19. This passive accumulation is further enhanced as nanoassemblies are captured within the disrupted tight junctions between hepatocytes during the onset of ALF. Subsequently, the cathepsin B overexpressed in hepatocytes injured by exposure to APAP triggers a burst release of mesalamine from the system, alleviating drug-induced ALF through the inhibition of inflammatory responses associated with MAPK signaling pathways **(Scheme 1C)**. Meanwhile, SPNs minimize the potential risk of side effects from nonspecific drug exposure by remaining an inactive state in healthy hepatocytes due to the relatively low expression of cathepsin B **(Scheme 1D)**. Collectively, this study provides a valuable methodology for the repositioning of mesalamine into ALF treatment by developing it as a nanomedicine through a supramolecular assembly mechanism.

## Results and Discussion

### Physicochemical characterization

**Figure 1:**
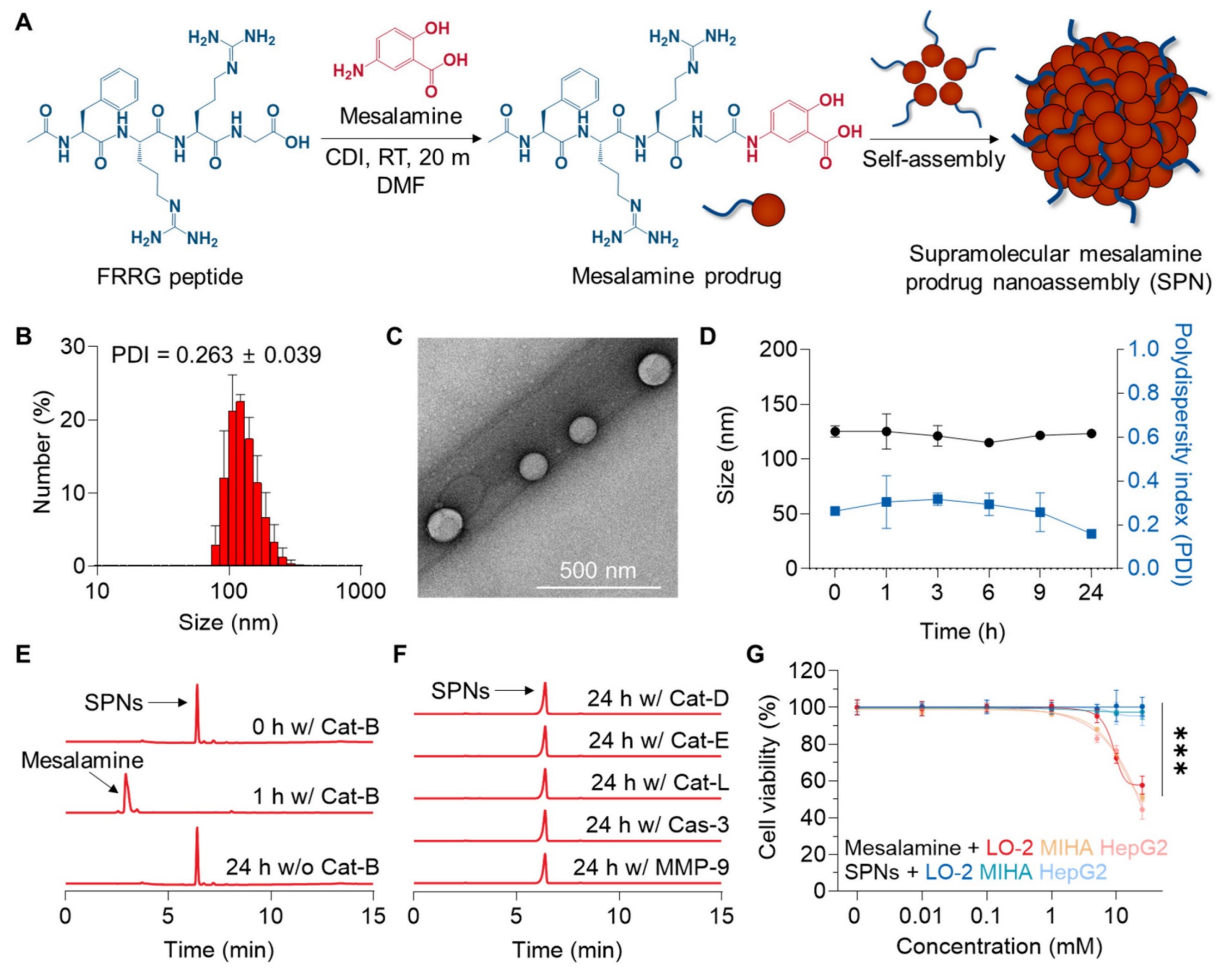
** Preparation and physicochemical characterization of SPNs. (A)** Synthetic diagram of the mesalamine prodrug. **(B)** Hydrodynamic size and **(C)** morphology of SPNs in an aqueous condition.** (D)** The size and polydispersity index (PDI) of SPNs in saline for 24 h. The cleavage behavior of SPNs when incubated with **(E)** Cat-B or **(F)** other enzymes. **(G)** Cell viability of LO-2, MIHA and HepG2 cells following treatment with mesalamine of SPNs. ***P < 0.001; significance was determined by one-way ANOVA with Tukey's post hoc test (G).

SPNs were self-assembled from a mesalamine prodrug, which was synthesized by conjugating mesalamine with the FRRG peptide through a one-step amide condensation reaction **(Figure 1A)**. Mesalamine was chosen for downregulating MAPK pathways in the livers and ultimately recovering ALF, as (i) no JNK inhibitors are currently available for clinical use, (iii) those inhibitors in preclinical studies lack a reactive group suitable for chemical modification with the FRRG peptide 20 and (iii) mesalamine has been used in clinical practice for an extended period and is therefore expected to minimize concerns about potential toxicity. Following the reaction, mesalamine prodrug was obtained with over 99% purity, followed by verification of successful synthesis by observing their calculated molecular weight **(**712.6 m/z [M] and 356.9 m/z [M+2H]^2+^; **Figure S1)**. These conjugates spontaneously formed nanoassemblies with spherical morphology and hydrodynamic size of 125 ± 5.2 nm under aqueous conditions **(Figure 1B-C)**. Hence, SPNs consist of 100% prodrugs with a drug loading content of 21.6%, which is higher than that of conventional nanoparticles, such as polymeric nanoparticles (≈ 10%) and lipid nanoparticles (≈ 5%) 21, 22. We have previously identified the self-assembly mechanism of this conjugate as hydrogen bonds and π-π stacking interactions between mesalamine and the aromatic segment of phenylalanine, respectively 14. The zeta potential was determined to be + 23.13 ± 1.91 mV. SPNs exhibited outstanding stability in saline, with no significant changes in average size and polydispersity index (PDI) for 24 h of incubation **(Figure 1D and S2)**. Such stable nanoparticles, approximately 100 nm in size, are suitable for expecting considerable accumulation within the liver through its 100-150 nm vascular fenestrations 19. Since a variety of targeting mechanisms can be expected, nanoparticles have long been recognized as a promising technology with huge potential in diagnostics and treatment for various diseases 23-25. The nanoassemblies were completely decomposed into mesalamine upon incubation with Cat-B for 1 h in pH 5.5 MES buffer **(Figure 1E)**. LC/MS analysis supported this result, confirming the molecular weight of mesalamine (calculated mass: 153.14 Da; measured mass: 154.1 m/z [M+H]) at the peak in the HPLC spectrum that emerged after incubation with Cat-B **(Figure S3)**. In contrast, no detectable cleavage was observed after 24 h of incubation when SPNs were exposed to other enzymes, such as Cat-D, Cat-E, Cat-L, Caspase 3 (Cas-3) and matrix metalloproteinase 9 **(**MMP-9; **Figure 1F)**. With this target enzyme-specificity, SPNs significantly minimized the toxicity of mesalamine to hepatocellular carcinoma HepG2 cells and human hepatocytes LO-2 and MIHA cells **(Figure 1G)**. Although mesalamine is generally well tolerated, various adverse events have been reported, including worsening colitis, interstitial nephritis and pulmonary toxicity 26. Based on these results, we postulated that SPNs may exhibit superior efficacy through a burst release of active compounds specifically in injured hepatocytes, while reducing side effects by minimizing nonspecific drug release at off-target sites.

### MAPK inhibition in hepatocytes

**Figure 2:**
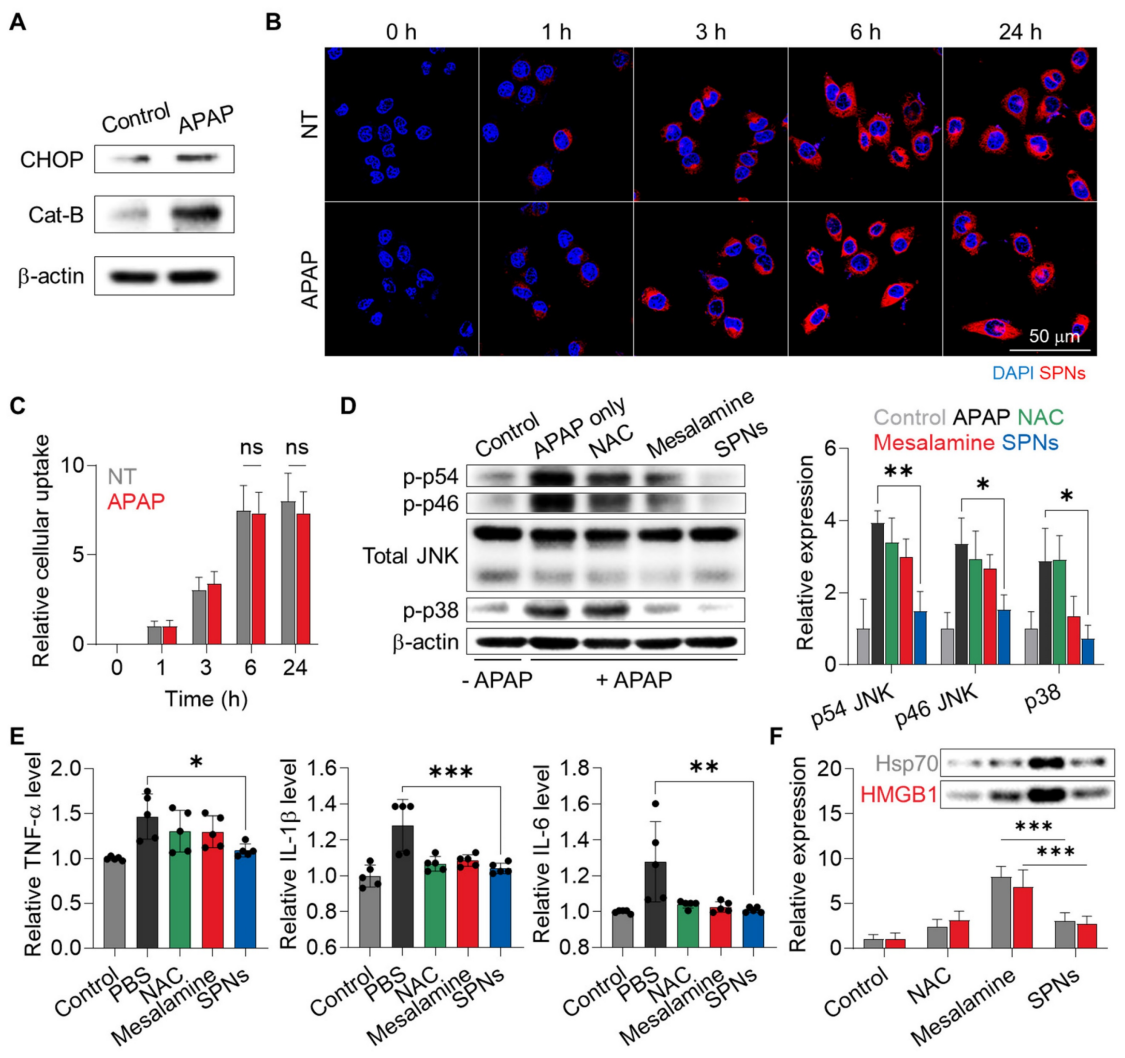
** Cellular uptake and MAPK inhibition of SPNs in hepatocytes. (A)** Expression of CHOP and Cat-B in LO-2 cells before and after treatment with APAP. **(B)** Representative confocal microscopy images of naive and APAP-treated LO-2 cells after treatment with Cy5.5-SPNs. **(C)** The quantity of Cy5.5-SPNs in naive and APAP-treated LO-2 cells after varying incubation time. **(D)** Expression levels of total and phosphorylated JNKs and p38 in APAP-treated LO-2 cells and those cells treated with NAC, SPNs or mesalamine for 6 h. **(E)** Levels of cytokines in APAP-treated LO-2 cells and those cells treated with NAC, SPNs or mesalamine for 6 h. **(F)** Release of DAMPs from LO-2 cells after treatment with NAC, mesalamine or SPNs. *P < 0.05, ***P < 0.001; significance was determined by one-way ANOVA with Tukey's post hoc test (D, E, F).

We initially investigated the changes within human hepatocytes in response to drug exposure. The LO-2 cells exhibited 4.22-fold higher levels of Cat-B, along with a 1.58-fold increase in C/EBP homologous protein (CHOP), which is involved in MAPK-mediated apoptosis and inflammation, after treatment with APAP compared to the naive cells **(Figure 2A and S4)**. These results indicate that hepatocytes significantly overexpress Cat-B following injury due to inflammatory responses associated with MAPK.

To assess the cellular uptake of SPNs, 1 wt% of Cy5.5-labeled mesalamine prodrug was co-assembled with 99% unmodified prodrugs **(Figure S5)**. When the naive or APAP-treated LO-2 cells were incubated with Cy5.5-SPNs, robust cellular uptake was observed, with no significant difference in uptake before and after APAP treatment **(Figure 2B)**. The amount of nanoassemblies within the hepatocytes gradually increased in a time-dependent manner, plateauing at 6 h post-incubation **(Figure 2C)**. Afterward, the effects of FRRG-ASA in inhibiting MAPK were evaluated in LO-2 cells. Incubation with APAP significantly upregulated the levels of phosphorylated JNKs and p38 related to MAPK signaling pathways in hepatocytes **(Figure 2D and S6)**. Importantly, subsequent treatment with SPNs resulted in a notable decrease in these inflammatory mediators in APAP-treated LO-2 cells cells after 6 h of incubation, with levels of p54 JNK, p46 JNK and p38 decreasing to 37.7%, 45.39% and 25.18%, respectively, recovering to levels similar to the basal levels in untreated cells. Lastly, a significant decrease in the levels of proinflammatory cytokines, such as tumor necrosis factor-alpha (TNF-α), interleukin-1 beta (IL-1β) and IL-6, was observed in APAP-treated LO-2 cells after 6 h of treatment with SPNs **(Figure 2E)**. The effects of SPNs in inhibiting MAPK and inflammatory cytokines in vitro were nearly comparable to those of the parent drug or N-acetylcysteine (NAC), a clinically approved drug to prevent ALF induced by APAP overdose 27. However, healthy LO-2 cells released significantly higher levels of damage-associated molecular patterns (DAMPs), such as high mobility group box 1 (HMGB1) and heat shock protein 70 (HSP70), after treatment with mesalamine compared to NAC and SPNs **(Figure 2F)**. Importantly, treatment with SPNs did not increase DAMP release in hepatocytes, which can be attributed to the sustained release of mesalamine at a constant level due to the prodrug system, thereby preventing excessive intracellular accumulation. Similar beneficial effects were further observed in HepG2 cells incubated with H_2_O_2_, which is commonly used to induce oxidative stress for intuitive modeling **(Figure S7)**.

Next, we evaluated the effects of SPNs on macrophage polarization. Lipopolysaccharide (LPS)-stimulated RAW 264.7 cells exhibited a pro-inflammatory M1 phenotype, characterized by low CD206 and high inducible nitric oxide synthase **(**iNOS; **Figure S8)**. Subsequent treatment with nanoassemblies induced macrophage polarization from an M1 to an M2 phenotype, with SPNs significantly upregulating CD206 expression and downregulating iNOS in LPS-treated RAW 264.7 cells. These results demonstrate their potential role in modulating the inflammatory environment in ALF by inducing anti-inflammatory phenotypic changes in Kupffer cells, the liver-resident macrophages.

### Enhanced liver accumulation in an ALF mouse model

**Figure 3:**
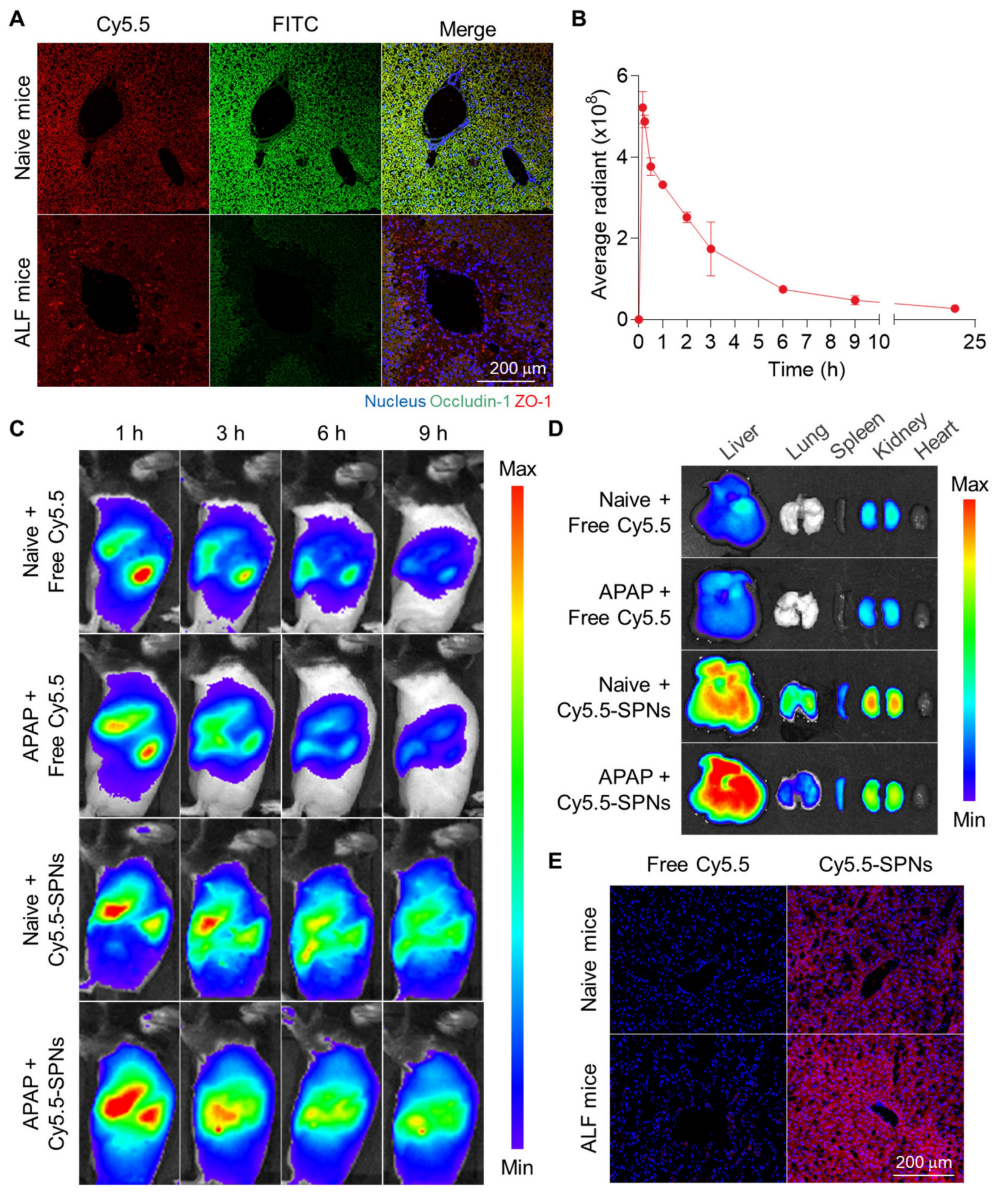
** Enhanced liver accumulation in an ALF mouse model. (A)** Representative confocal microscopy images of sectioned liver tissues from naive and ALF mice after staining with ZO-1 and occludin-1 antibodies. **(B)** Pharmacokinetic (PK) profile of intravenously injected SPNs in ALF mice. **(C)** Whole-body NIRF images of naive and ALF mice after intravenous injection of free Cy5.5 or Cy5.5-SPNs. **(D)** *Ex vivo* NIRF images of major organs.** (E)** Representative confocal microscopy images of liver tissues after 9 h of intravenous injection of free Cy5.5 or Cy5.5-SPNs.

The enhanced liver accumulation of SPNs due to the disruption of tight junctions was evaluated in an ALF model established by intraperitoneal administration of APAP in C57BL/6 mice. The extensive destruction of tight junctions between hepatocytes within the liver tissues was confirmed in mice after APAP injection, as evidenced by the loss of two major tight junction-associated proteins, occludin-1 and zonula occludens-1 (ZO-1), compared to naive mice **(Figure 3A)**. These findings show that drug-induced hepatotoxicity causes tight junction disruption in livers, leading to a pathological environment where nanoparticles can accumulate passively.

Afterward, the pharmacokinetic (PK) profile of intravenously injected SPNs was investigated in ALF mice, revealing prolonged circulation in vivo, with detectable levels observed in the blood for 24 h **(Figure 3B)**. The biodistribution of SPNs was then monitored *via* near-infrared fluorescence (NIRF) imaging. Free Cy5.5 was employed as a small molecule model drug for mesalamine, which has a molecular weight too low to directly incorporate a fluorescent dye. Following intravenous injection, NIRF images of ALF mice revealed a significant accumulation of Cy5.5-SPNs in the liver (black dotted line) compared to free Cy5.5 **(Figure 3C)**. Fluorescence signals from nanoassemblies within the liver tissues were sustained for 9 h post-treatment, in stark contrast to free Cy5.5, which exhibited rapid *in vivo* clearance due to small molecule structure. Interestingly, the hepatic accumulation of Cy5.5-SPNs in ALF models was greater compared to the Sham group; in contrast, Cy5.5 showed similar behavior in both mouse models. These results indicate that nanoassemblies can accumulate in liver tissues through interstices resulting from the breakdown of tight junctions during the onset of ALF, due to their nano-sized particles, enabling efficient drug delivery. *Ex vivo* NIRF images of major organs further confirmed a substantial accumulation of Cy5.5-SPNs in liver tissues from ALF mice compared to naive mice, showing a 22-fold higher fluorescence intensity relative to free Cy5.5 at 9 h post-administration **(Figure 3D and S9)**. Histological analysis demonstrated that the strong fluorescence signals from Cy5.5-SPNs were evenly distributed in the liver tissues **(Figure 3E and S10)**.

### Therapeutic efficacy of SPNs in an ALF model

**Figure 4:**
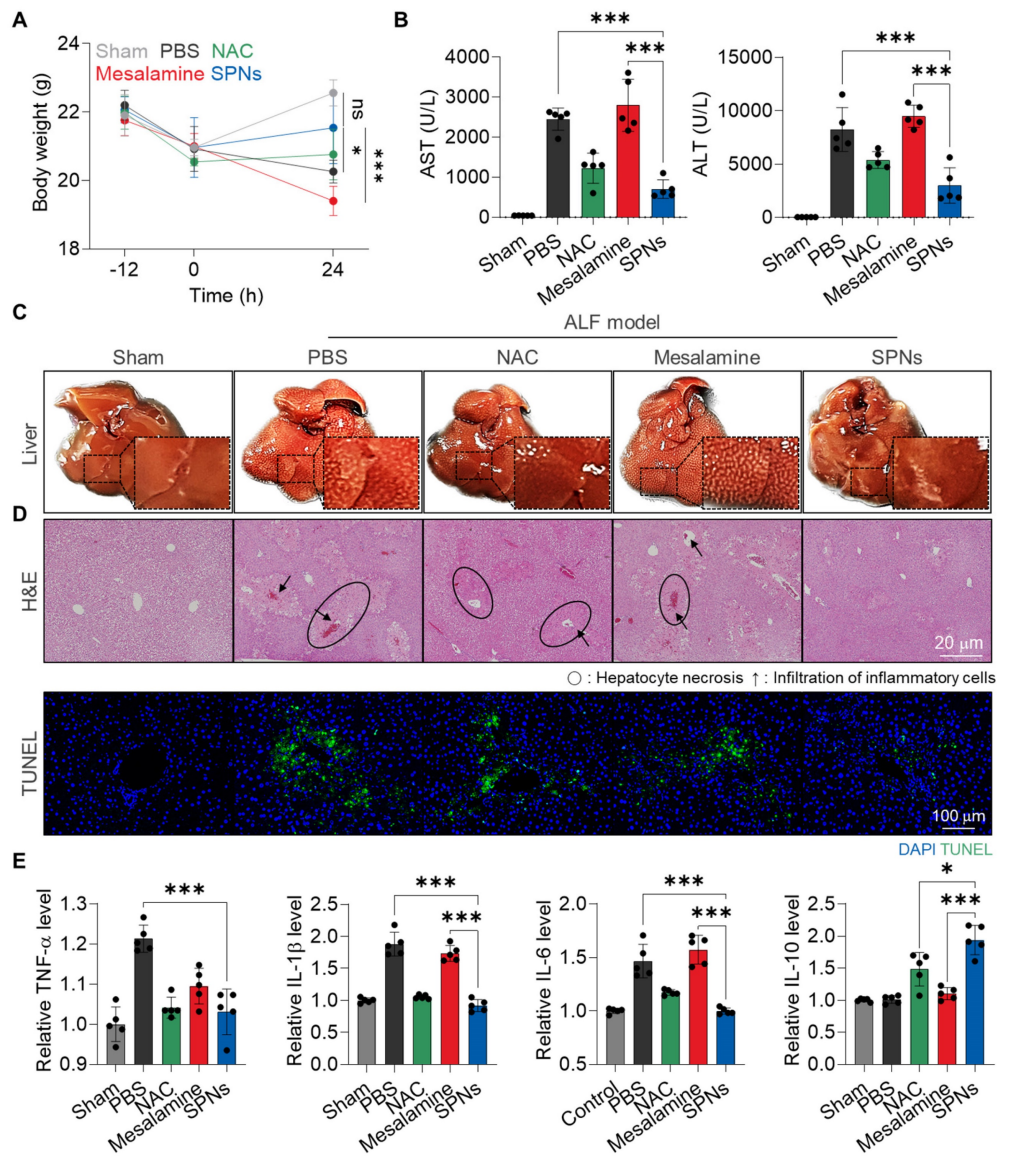
** Therapeutic efficacy of SPNs in an ALF model. (A)** Changes in body weight in APAP-induced ALF mice after treatment with intravenous injection of PBS, NAC, mesalamine or SPNs, compared to healthy mice.** (B)** Levels of hematological parameters related to hepatic toxicity in ALF mice at 24 h post-treatment with PBS, NAC, mesalamine or SPNs.** (C)** Optical images of liver tissues and **(D)** H&E- or TUNEL-stained sections.** (E)** Levels of cytokines in liver tissues. *P < 0.05, ***P < 0.001; significance was determined by one-way ANOVA with Tukey's post hoc test (A, B, E).

The *in vivo* therapeutic efficacy of SPNs was evaluated in an APAP-induced ALF model. For these analyses, C57BL/6 mice were randomly divided into five groups: (i) Sham, (ii) APAP + PBS (PBS), (iii) APAP + NAC, (iv) APAP + mesalamine (Mesalamine) and (v) APAP + SPNs (SPNs). The mice were intraperitoneally injected with APAP and treated with mesalamine or SPNs (5 mg/kg based on mesalamine content) at 3 h after APAP administration **(Figure S11)**; as a control, NAC is intravenously injected at a dose of 100 mg/kg 28. SPNs effectively downregulated MAPK in hepatic tissues compared to mesalamine and NAC, resulting in significant effects on ALF recovery **(Figure S12)**. First, treatment with SPNs significantly protected the animals from body weight loss due to APAP-induced hepatotoxicity, showing no significant changes compared to the Sham group after 24 h of treatment **(Figure 4A)**. In contrast, mice in the mesalamine group exhibited even more severe weight loss relative to the PBS group, attributed to its toxic side effect. Hematological parameters related to hepatic toxicity, such as aspartate aminotransferase (AST) and alanine transaminase (ALT), were also elevated in mice following APAP injection, but subsequent treatment with SPNs noticeably reduced these levels to those similar to the Sham group **(Figure 4B)**. As expected, the mesalamine group showed higher levels of these parameters compared to the PBS group. Morphological and histopathological changes, along with apoptotic cell death, in liver tissues were observed in the PBS and mesalamine groups, whereas these alterations were minimized in the SPNs group **(Figure 4C-D)**. NAC treatment showed limited effects in preventing body weight loss and histohematological changes than SPNs. Lastly, enzyme-linked immunosorbent assays (ELISAs) and histology indicated that SPN treatment reduced the levels of upregulated proinflammatory cytokines in liver tissues following APAP administration and significantly increased the quantity of the anti-inflammatory cytokine IL-10 **(Figure 4E and S13)**. These findings underscore the outstanding therapeutic efficacy of nanoassemblies in delivering mesalamine efficiently and selectively to injured hepatocytes.

**Figure 5:**
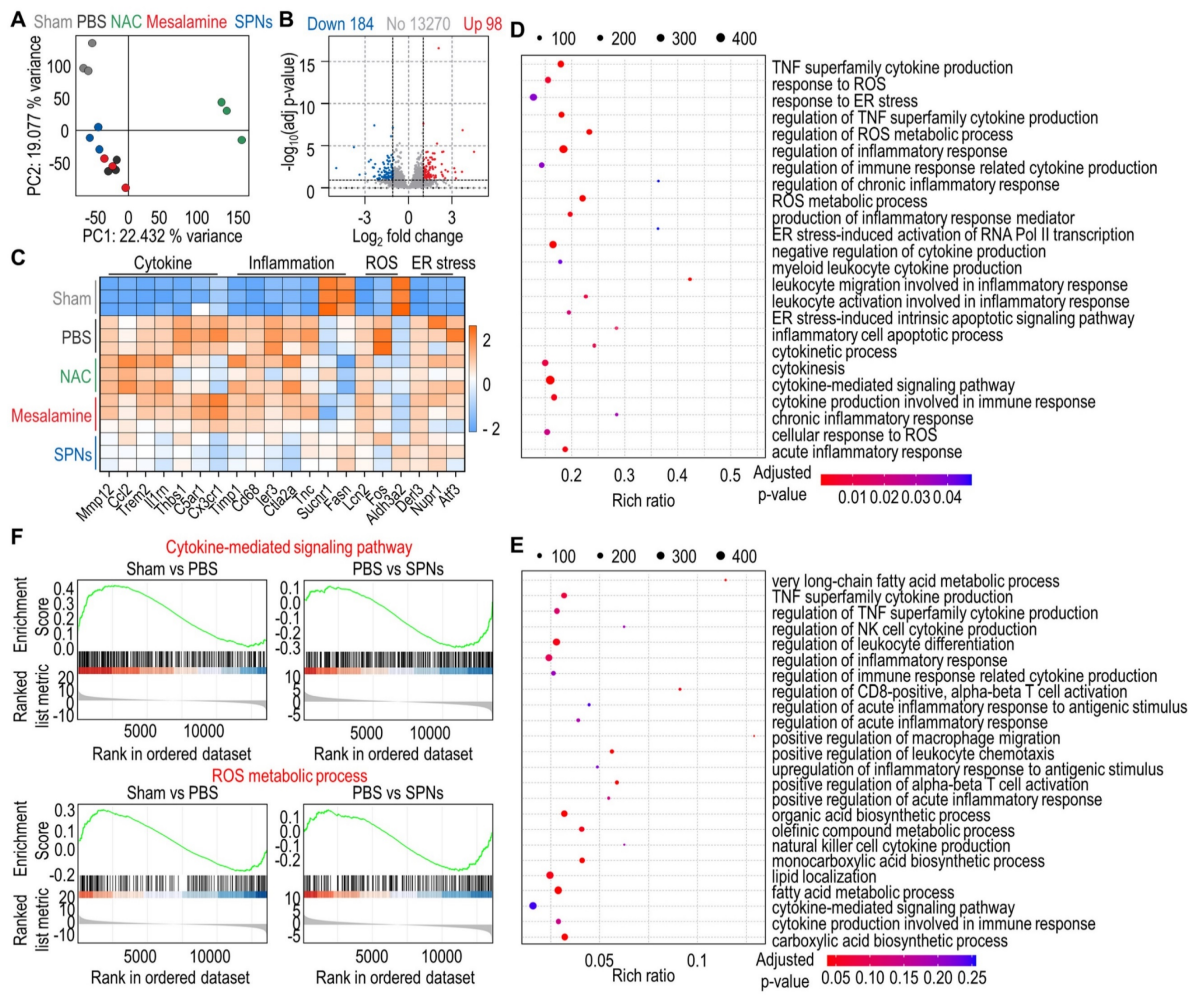
** Transcriptomic analysis by RNA-seq. (A)** PCA plots based on the DEGs from naive mice and ALF mice treated with PBS, NAC, mesalamine or SPNs. **(B)** Volcano plot of the DEGs between the PBS and SPNs groups. **(C)** Heatmap of the top 20 DEGs in each group. **(D-E)** KEGG enrichment analysis of pathways associated with MAPK in DEGs between the PBS and SPNs groups. **(F)** GSEA of the DEGs related to the cytokine-mediated signaling pathway and ROS metabolic process in the liver, comprising the PBS vs SPNs group.

To provide more insight into the underlying therapeutic mechanism of SPNs against ALF, RNA sequencing was performed on total RNA extracted from liver tissues. Principal component analysis (PCA) revealed an obvious difference in distribution characteristics between the PBS and SPN groups **(Figure 5A)**. Additionally, volcano plots identified a total of 282 differentially expressed genes (DEGs) between the PBS and SPNs groups, including 98 upregulated genes and 184 downregulated genes **(Figure 5B)**. The heatmap further displayed these DEGs across the five groups, showing that ALF mice treated with SPNs exhibited noticeably reduced expression of genes related to cytokines, inflammation, ROS and ER stress compared to those treated with PBS, NAC or mesalamine **(Figure 5C)**. As illustrated in the bubble diagram, the genes related to MAPK signaling pathways were more enriched in the PBS group than in the SPN group **(Figure 5D-E)**. Lastly, gene set enrichment analysis (GSEA) showed that the cytokine-mediated signaling pathway and ROS metabolic process were found to be upregulated in ALF mice relative to naive mice, but treatment with SPNs significantly reduced the expression of genes associated with these pathways **(Figure 5F)**. In brief, SPNs mitigate inflammatory responses in the livers of ALF mice by downregulating MAPK signaling pathways, consistent with the aforementioned results.

### Safety of SPN treatment

**Figure 6:**
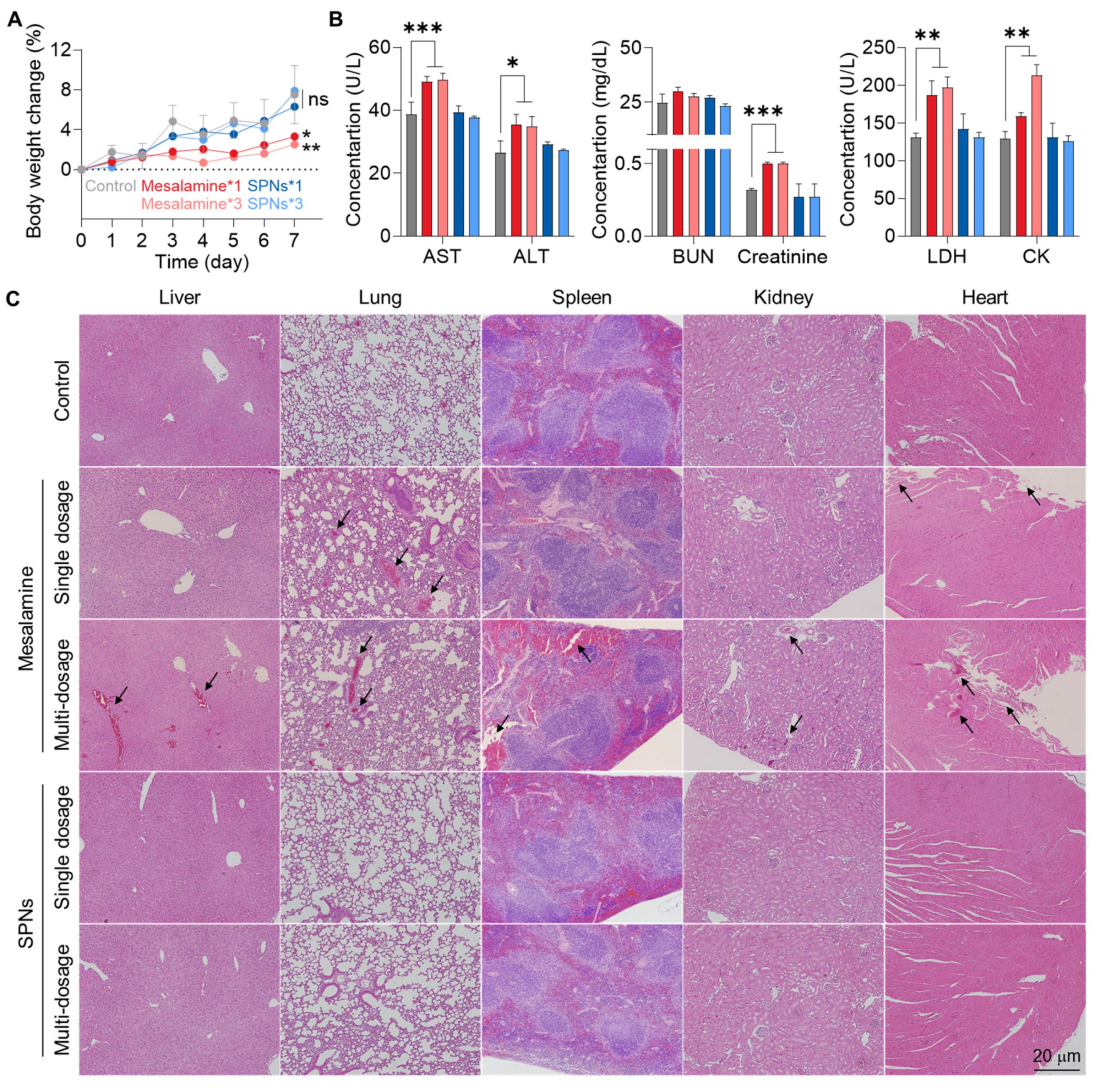
***In vivo* safety of SPNs. (A)** Body weight change in mice after treatment with a high dose of mesalamine or SPNs at single or multiple dosages (three times daily).** (B)** Levels of hematological parameters related to hepatic, renal and cardiac toxicities.** (C)** Major organs stained with H&E with black arrows indicating structural abnormalities. *P < 0.05, **P < 0.01, ***P < 0.001; significance was determined by one-way ANOVA with Tukey's post hoc test (A, B).

The *in vivo* safety of SPNs was evaluated in normal C57BL/6 mice, where mesalamine or SPNs were intravenously injected at a concentration two times higher than the dose used in therapeutic efficacy studies, with single or multiple dosages (three times daily). No significant changes in body weight were observed in any of the SPN groups compared to the untreated control group **(Figure 6A)**. In contrast, administration of mesalamine caused significant body weight loss, which became more severe with an increased number of doses. Several hematological parameters indicated severe hepatic (AST and ALT), renal (blood urea nitrogen (BUN) and creatinine) and cardiac (lactate dehydrogenase (LDH) and creatinine kinase (CK)) toxicities in the mesalamine groups **(Figure 6B and S14)**. However, those parameters in the SPN groups were within the normal range, similar to the untreated control group. There were no significant alterations in routine blood components across all mesalamine and SPN groups **(Figure S15)**. We also observed a considerable increase in inflammatory cytokine levels in the kidneys of mice following treatment with single and multiple doses of mesalamine, whereas these levels in both SPN groups were nearly comparable to those in the control group **(Figure S16)**. Although intravenously administered nanoassemblies were retained for a long time in major organs as shown in **Figure 3**, mesalamine-induced hepatic, cardiac and renal toxicities were mitigated by maintaining an inactive state, which was supported by the significantly lower expression of cat-B in healthy organs compared to liver tissues exposed to APAP **(Figure S17)**. In agreement with the above results, mesalamine treatment resulted in substantial structural abnormalities and damaged areas in major organs, but these histopathological toxicities were not observed in the SPN group **(Figure 6C)**. Overall, these results suggest that SPNs exhibit excellent therapeutic efficacy while remaining in an inactive state at off-target sites, thereby minimizing the adverse events associated with mesalamine.

## Conclusion

In summary, this study aimed to develop an innovative formulation to repurpose mesalamine, a medication used for treating colitis in clinical settings, for the treatment of ALF. First, we identified cathepsin B as an ALF biomarker for selectively delivering mesalamine to the target site and developed a mesalamine prodrug using its substrate peptide. By promoting the self-assembly of this prodrug *via* intermolecular interactions, the resulting supramolecular nanoassembly enabled passive accumulation in hepatic tissues through the interstices that occurred by the destruction of tight junctions during the onset of ALF. The most significant benefit is the reduction of mesalamine's toxic side effects due to cathepsin B-mediated selective drug release. Considering mesalamine's broad anti-inflammatory mechanism, this approach could inspire drug repositioning research not only for ALF but also for various inflammatory diseases. From an industrial perspective, its simple structure also offers the important advantage of being highly amenable to mass production and quality control, similar to small molecule drugs. By utilizing microfluidic technology, we expect to achieve a more uniform and scalable preparation of nanoparticles in future studies. As an additional potential indication, we are now evaluating the effects of SPNs in treating acute kidney injury (AKI), which is considered highly refractory due to the lack of effective therapeutic options. Ultimately, this study provides a rational design for nanomedicine to reposition mesalamine for effective and safer treatment of ALF.

## Materials and Methods

### Preparation and characterization of SPNs

To prepare the mesalamine prodrug, N-terminal acetylated FRRG peptide (100 mg, 0.175 mmol, 1 eq; Peptron Co., Daejeon, Republic of Korea) was reacted with mesalamine (26.74 mg, 0.175 mmol, 1 eq; Tokyo Chemical Industry, Toshima, Tokyo, Japan) in the presence of carbonyldiimidazole (33.75 mg, 0.21 mmol, 1.2 eq). After a 30 min reaction, the final product was purified and analyzed using an LC/MS system (Agilent 1200 Series HPLC system). The hydrodynamic size and zeta potential were analyzed by using a Zetasizer (Nano ZS, Malvern Instruments, Worcestershire, UK). The morphology of SPNs in distilled water was observed using transmission electron microscopy (CM-200, Philips, USA). The target enzyme-specific cleavage was evaluated through HPLC analysis after incubation with Cat-B (953CY, R&D systems, MN, USA), Cat-E (1294-AS, R&D systems), Cat-D (1014-AS, R&D systems), Cat-L (952-CY, R&D systems), MMP-9 (911-MP, R&D systems) or Cas-3 (707-C3/CF, R&D systems) in pH 5.5 2-(N-morpholino)ethane sulfonic acid (MES) buffer at 37°C.

### Cellular uptake in hepatocytes

The cellular uptake of SPNs was assessed in the hepatocellular carcinoma cell line HepG2 and the human hepatocyte cell line LO-2 (American Type Culture Collection, Manassas, VA, USA). For fluorescence imaging, Cy5.5-labeled mesalamine prodrug was prepared and co-assembled with an unmodified prodrug. Briefly, Cy5.5 NHS ester (50 mg, 0.065 mmol; Lumiprobe, Wan Chai, Hong Kong), NH_2_-FRRG-COOH peptide (59.42 mg, 0.13 mmol; Peptron Co.) and N, N diisopropylethylamine (10.06 mg, 0.078 mmol; Sigma Aldrich, St. Louis, MO, USA) were reacted in anhydrous dimethylformamide. Following 3 h of incubation, the resulting Cy5.5-FRRG-COOH was purified using Sep-Pak C18 cartridges (Waters, Massachusetts, USA), and its conjugation with mesalamine was conducted using the same protocol described above. Thereafter, Cy5.5-labeled mesalamine prodrug and unmodified prodrug were dispersed in saline at a ratio of 1:99 wt%. The HepG2 cells were incubated with 300 μM H_2_O_2_ for 4 h and subsequently treated with Cy5.5-SPNs. The cells were then incubated with fixatives for 10 min and stained with DAPI solution (Invitrogen, Carlsbad, CA, USA) for 10 min under dark conditions. NIRF imaging was performed with confocal laser scanning microscopy. The fluorescence intensity in the images was quantified using ImagePro Plus software (Media Cybernetic, Rockville, USA). For the cytotoxicity assays, LO-2, MIHA and HepG2 cells were treated with mesalamine or SPNs (0 to 20 mM) for 24 h, followed by additional incubation with 10% CCK-8 solution.

### MAPK analysis

The MAPK-inhibitory effect was assessed in APAP (20 mM for 4 h)-treated LO-2 cells and H_2_O_2_ (300 μM for 4 h)-treated HepG2 cells after treatment with NAC, SPNs or mesalamine (1 mM) for 6 h. The cells were then washed with DPBS and dispersed in lysis buffer, followed by centrifugation at 12,000 rpm for 30 min to remove cell debris. Subsequently, the proteins were quantified using a BCA assay kit (23221, Thermo Fisher Scientific), boiled after mixing with sodium dodecyl sulfate loading buffer (NaraBio, Gyeonggi-do, Republic of Korea) for 5 min, and separated by 10% SDS polyacrylamide gel electrophoresis. Following transfer onto polyvinylidene fluoride membranes, they were incubated with rabbit anti-mouse antibodies targeting total (#9252, Cell Signaling)/phosphorylated JNKs (MAB1205, R&D systems), p38 (ab195049, Abcam, Cambridge, UK), HMGB1 (ab18256, Abcam), HSP70 (ab2787, Abcam) or β-actin (ab8227, Abcam) at 4 °C for 24 h. HMGB1 and HSP70 were analyzed in the supernatants after incubating LO-2 cells using the same protocol described above. Finally, the membranes were incubated with goat anti-rabbit immunoglobulin-HRP antibody for 2 h. The cytokines, including TNF-α (43907, San Diego, CA, USA), IL-6 (M6000B, R&D systems), IL-10 (M1000B-1, R&D systems) and IL-1β (MLB00C, R&D systems), in H_2_O_2_-treated HepG2 cells were assessed after treatment with SPNs or mesalamine (1 mM) for 6 h.

### Macrophage phenotypic change

Macrophage polarization was analyzed in LPS-stimulated RAW 264.7 cells following treatment with SPNs or mesalamine at a concentration of 1 mM for 24 h. LPS (L2630, Sigma Aldrich) was applied at 1 μg/mL for 5 h. The cells were then lysed and centrifuged at 12,000 rpm for 15 min to remove debris. Proteins, quantified using a bicinchoninic acid assay (BCA, 23221, Thermo Fisher Scientific), were boiled for 5 min after mixing with SDS loading buffer and separated on a 10% SDS-polyacrylamide gel. After transfer onto nitrocellulose (NC) membranes, the membranes were incubated with rabbit anti-mouse antibodies targeting CD206 (ab64693, Abcam) and iNOS (ab15323, Abcam) for 24 h at 4 °C, followed by incubation with goat anti-rabbit horseradish peroxidase (HRP)-tagged antibody (7074P2, Cell Signaling) at room temperature for 2 h. The HRP signal intensity on the membranes was visualized using enhanced chemiluminescence (ECL). The uncropped original images of all Western blot results were shown in **Figure S18**.

### Biodistribution and therapeutic efficacy

All experiments involving animals were conducted in accordance with relevant laws and institutional guidelines of the Institutional Animal Care and Use Committee (IACUC, approved number: 2023-013) at the Korea Institute of Science and Technology (KIST). The destruction of tight junctions within hepatocytes was assessed by comparing ZO-1 and occluding-1 in liver tissues from naive or ALF mice, which were prepared by intraperitoneal injection of 300 mg/kg APAP into 8-week-old male C57BL/6 mice. Liver tissues were collected from mice, followed by staining with Alexa Fluor 647-labeled antibody against ZO-1 (Cell Signaling, Massachusetts, USA) and Alexa Fluor 488-labeled antibody against occludin-1 (Invitrogen).

To assess the PK profile, Cy5.5-SPNs were intravenously administered into ALF mice based on a 5 mg/kg mesalamine content, followed by blood collection at the indicated time points. Blood samples were then centrifuged at 2,200 rpm for 20 min, and the resulting serum was subjected to protein precipitation by adding acetonitrile (1:3, v/v). Fluorescence intensity in the supernatants was quantified using a Lumina Series III (PerkinElmer, Waltham, MA, USA).

The *in vivo* behavior of SPNs was assessed in healthy mice and APAP-induced ALF models. Using Lumina Series III, NIRF imaging was conducted following intravenous injection with Cy5.5-SPNs (5 mg/kg based on mesalamine content) or free Cy5.5 with equivalent fluorescence intensity. Fluorescence intensity in the images was quantified using LivingImage software. *Ex vivo* NIRF imaging was conducted with major organs collected from the mice 9 h post-treatment.

To evaluate therapeutic efficacy, the ALF mouse model was intravenously injected with mesalamine or SPNs, at an equivalent concentration based on mesalamine content of 5 mg/kg, 3 h after APAP administration; as a control, NAC was administered intravenously at a dose of 100 mg/kg. Following 24 h of treatment, liver tissue was collected from the mice and then stained with H&E, fluorescent dye-conjugated antibodies (TNF-α, 506303, Biolegend; IL-6, 504503, Biolegend; and IL-10, 505005, Biolegend) or TUNEL (G3250, Promega). A portion of the liver tissues was dispersed in lysis buffer and subsequently centrifuged to remove debris, followed by western blot for MAPK and assessment of cytokines, such as TNF-α, IL-6, IL-1β and IL-10, *via* ELISA assays. Hematological parameters were analyzed by KNOTUS (Incheon, Republic of Korea).

### RNA sequencing analysis

The gene-level analysis was conducted by ATG Lifetech Inc. (Republic of Korea). Briefly, total RNA (10 ng) was extracted from liver tissues using S2 + Reverse-transcriptase (Cat# LP-T0012, ATG Lifetech Inc.), and RNA integrity was evaluated by analyzing the RNA Integrity Number (RIN). Complementary DNA (cDNA) was subsequently amplified using K1 HiFi PCR Master Mix (Cat# LP-T0013, ATG Lifetech Inc.). Tn5 transposase was employed to ligate next-generation sequencing (NGS) adapters to the cDNA, with only adapter-ligated fragments being amplified by the NGS Library Prep Kit (LP-T0011, ATG Lifetech Inc.). Preprocessing steps were performed on each sample using the NovaSeq 6000 with a 6G capacity and 150 bp paired-end (PE) sequencing.

### *In vivo* safety

For the analysis of *in vivo* safety, mesalamine or SPNs (10 mg/kg based on mesalamine content) were intravenously injected into 8-week-old male C57BL/6 mice at single or multiple dosages (three times daily). The body weight was measured every two days. Seven days after treatment, the hematological parameters were assessed, including AST, ALT, BUN, creatinine, LDH, CK, red blood cell (RBC), hemoglobin (HGB), hematocrit (HCT), platelet (PLT), white blood cell (WBC), neutrophil (NEU) and lymphocyte (LYM). Major organs were collected from the mice and then observed using optical microscopy after H&E staining (BX 51, Olympus, USA). The levels of TNF-α, IL-1β and IL-6 in homogenized kidney tissues were quantified via ELISA assays.

### Statistical analysis

Statistical significance between the two groups was analyzed using Student's t-test. In the case of more than two groups, one-way analysis of variance (ANOVA) was used, and multiple comparisons were performed using the Tukey-Kramer post-hoc test. Survival results were plotted using Kaplan-Meier curves and analyzed using the log-rank test. All results are presented as mean ± SD, and P values of < 0.05*, < 0.01** and < 0.001*** were considered statistically significant.

## Supplementary Material

Supplementary figures.
