# Supplementary material for: Drug repositioning of mesalamine via supramolecular nanoassembly for the treatment of drug-induced acute liver failure
Source: Theranostics. 2025 Jan 1;15(3):1122–34. doi: 10.7150/thno.101358 (PMC11700871; doi:10.7150/thno.101358)
Supplement: Supplementary file 1 — Supplementary figures. [file thnov15p1122s1.pdf]

Supplementary Information for

Drug repositioning of mesalamine via supramolecular nanoassembly for the treatment of drug-induced acute liver failure

Byeongmin Park[1,2], Eun Hye Kim[2,3], Hochung Jang[2,4], Yelee Kim[2,3], Youngri Ryu[2,3], Jiwoong Choi[2], Dongwon Shin[1,2], Myung Chul Lee[2], Yoosoo Yang[2,4], Kwangmeyung Kim[5], Sangmin Lee[6,*], Sun Hwa Kim[1,2,*], Man Kyu Shim[2,*]

[1]KU-KIST Graduate School of Converging Science and Technology, Korea University, Seoul, 02841, Republic of Korea.

[2]Medicinal Materials Research Center, Biomedical Research Division, Korea Institute of Science and Technology (KIST), Seoul, 02792, Republic of Korea.

[3]Department of Life Sciences, Korea University, Seoul 02841, Republic of Korea.

[4]Division of Bio-Medical Science and Technology, KIST School, Korea University of Science and Technology, Seoul 02792, Republic of Korea.

[5]College of Pharmacy, Graduate School of Pharmaceutical Sciences, Ewha Womans University, Seoul 03760, Republic of Korea.

[6]College of Pharmacy, Graduate School of Pharmaceutical Sciences, Kyung Hee University, Seoul 02453, Republic of Korea.

*Correspondence and requests for materials should be addressed to **M. K. Shim** (E-mail: mks@kist.re.kr), **S. H. Kim** (E-mail: sunkim@kist.re.kr) and **S. Lee** (E-mail: leesm@khu.ac.kr).

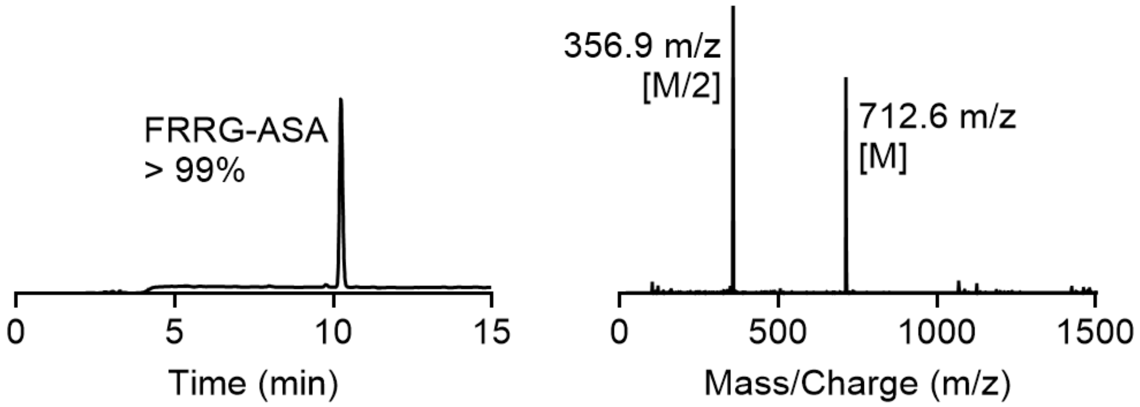

Figure S1. The purity and molecular weight of mesalamine prodrug, as confirmed by LC/MS.

	Incubation time in Saline					
	0 h	1 h	3 h	6 h	9 h	24 h
Size (nm)	125.3 ± 5.198	125.3 ± 16.18	121.1 ± 9.326	115.0 ± 2.052	121.6 ± 0.153	123.4 ± 3.808
PDI	0.263 ± 0.012	0.304 ± 0.121	0.317 ± 0.028	0.293 ± 0.051	0.258 ± 0.088	0.16 ± 0.02

Figure S2. Detail information on hydrodynamic size and polydispersity index (PDI) of SPNs upon incubation in saline for 24 h.

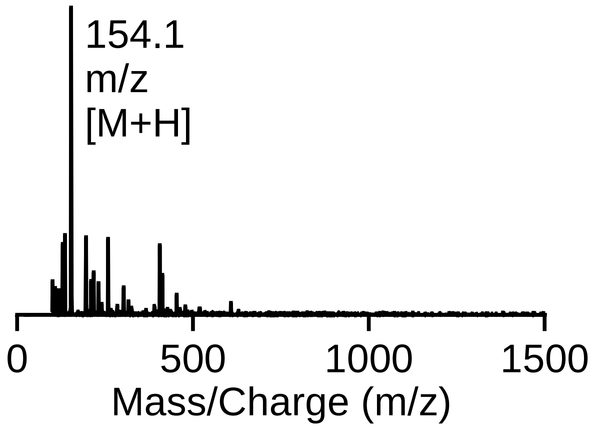

Figure S3. Mass analysis of emerged peak in the HPLC spectrum after incubation of SPNs cathepsin B in pH 5.5 MES buffer.

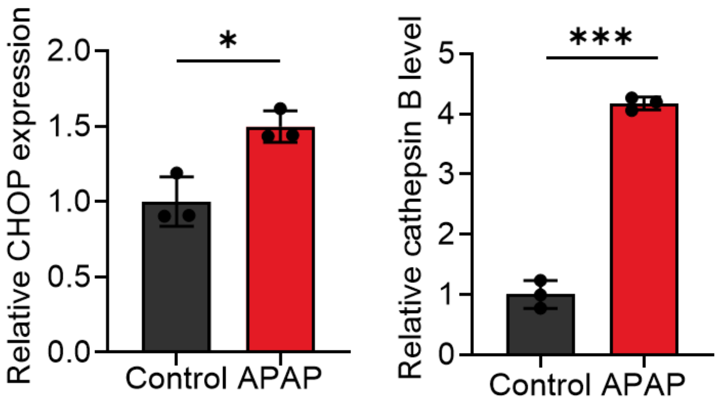

Figure S4. Quantitative analysis of CHOP and Cat-B in LO-2 cells before and after treatment with APAP.

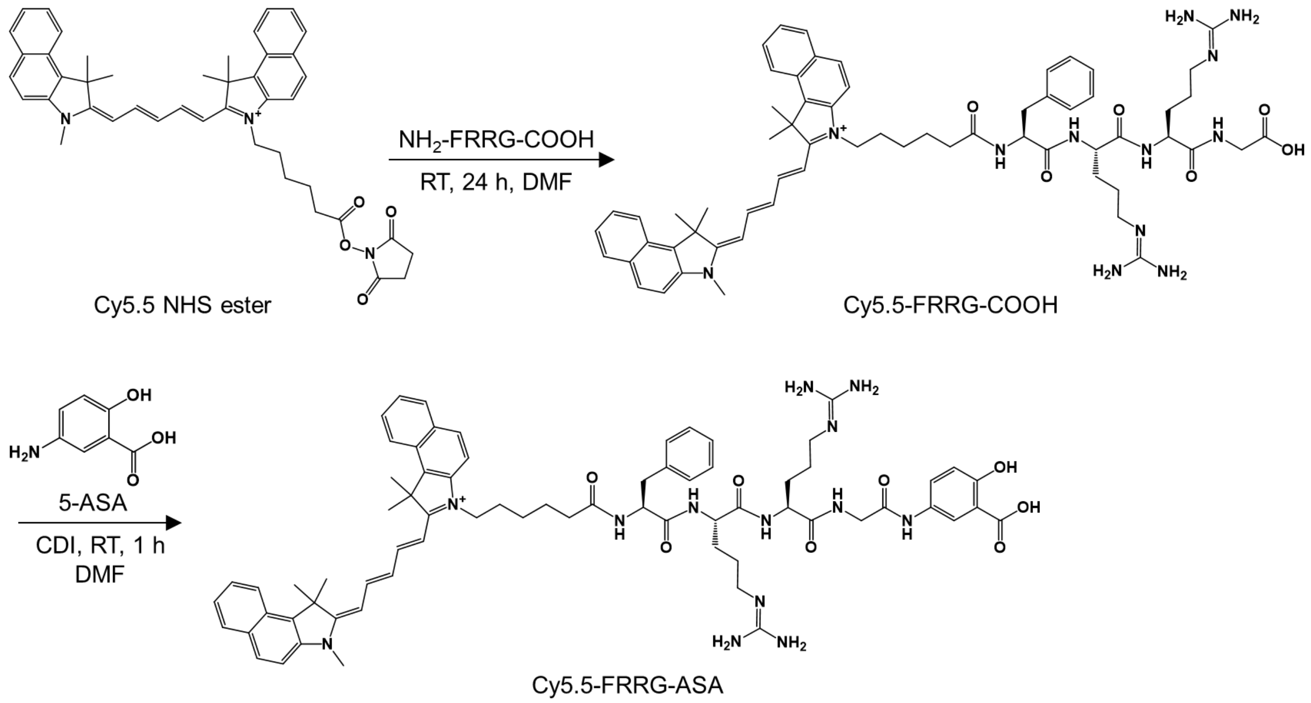

Figure S5. Synthetic diagram of Cy5.5-labeled mesalamine prodrug.

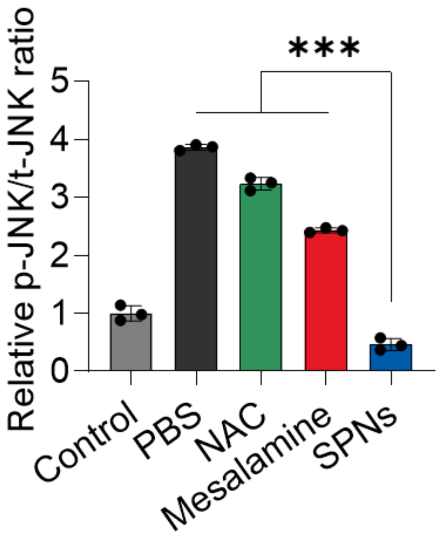

Figure S6. The relative ratio of phosphorylated JNK to total JNK levels in APAP-treated LO-2 cells and those cells treated with NAC, SPNs or mesalamine for 6 h.

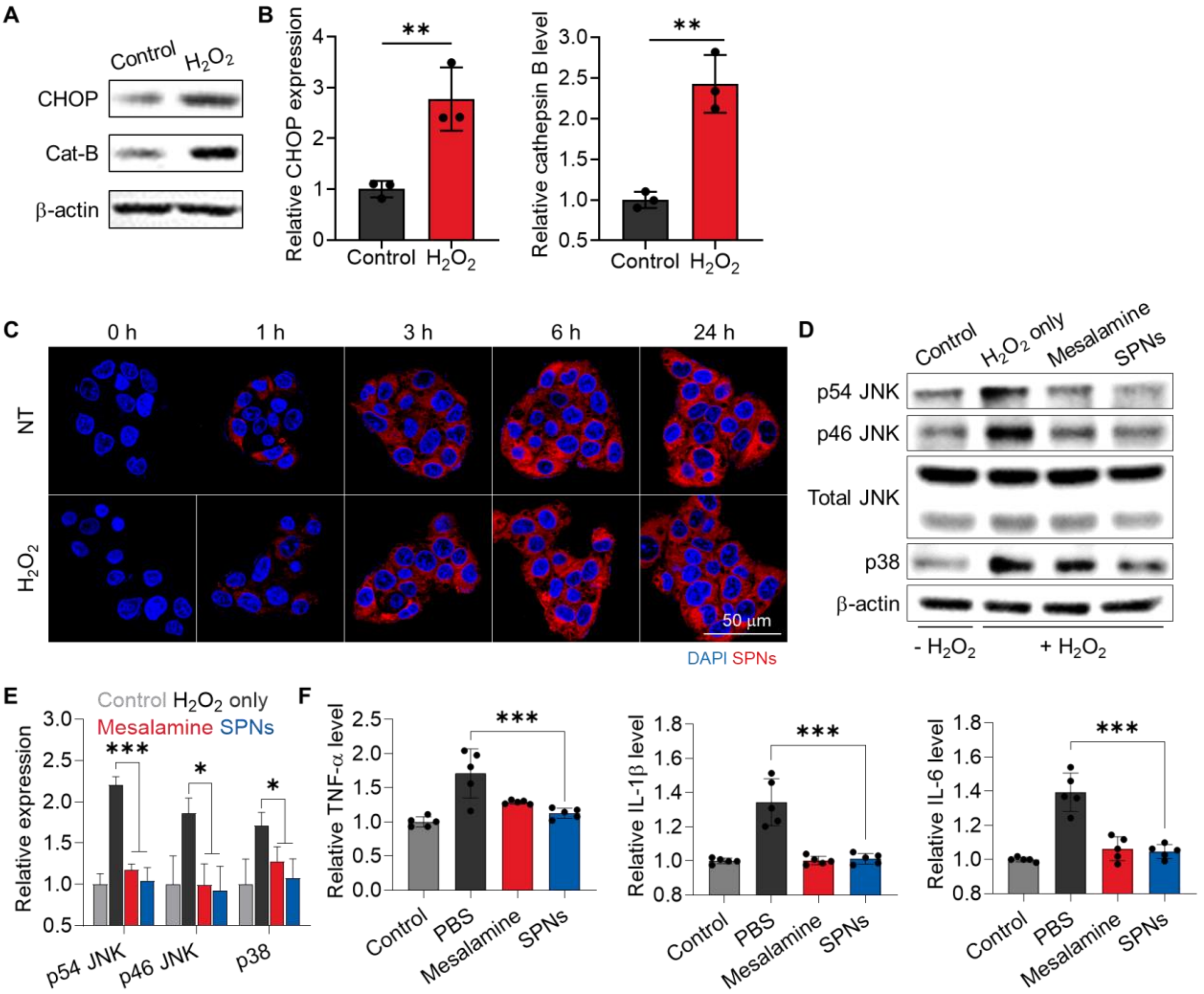

Figure S7. (A, B) Expression of CHOP and Cat-B in HepG2 cells before and after treatment with H_2O_2. **(C)** Representative confocal microscopy images of naive and H_2O_2-treated HepG2 cells after treatment with Cy5.5-SPNs. **(D, E)** Expression levels of phosphorylated JNKs and p38 in H_2O_2-treated HepG2 cells and those cells treated with SPNs or mesalamine for 6 h. **(F)** Levels of cytokines in H_2O_2-treated HepG2 cells and those cells treated with SPNs or mesalamine for 6 h.

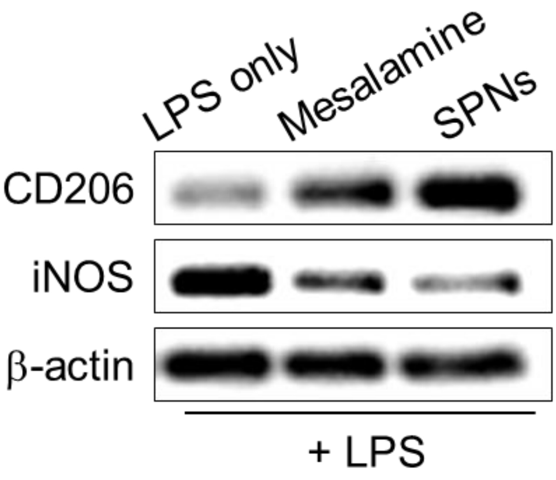

Figure S8. Expression levels of CD206 and iNOS in LPS-stimulated RAW 264.7 cells before and after treatment with mesalamine or SPNs.

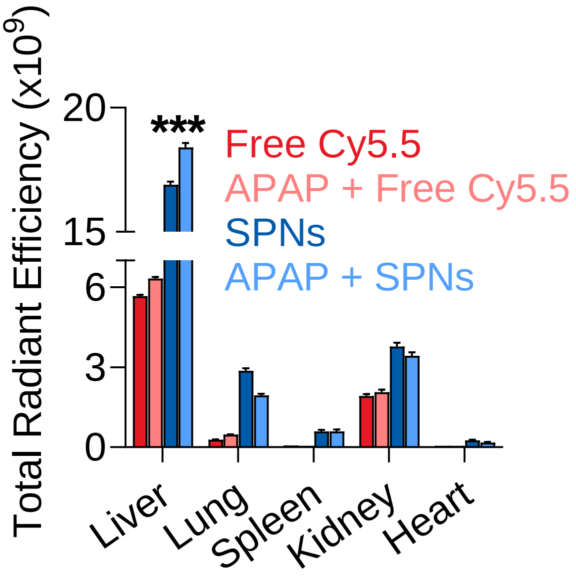

Figure S9. Quantitative analysis of fluorescence intensity of free Cy5.5 and SPNs in major organs from ALF mice following 9 h of treatment.

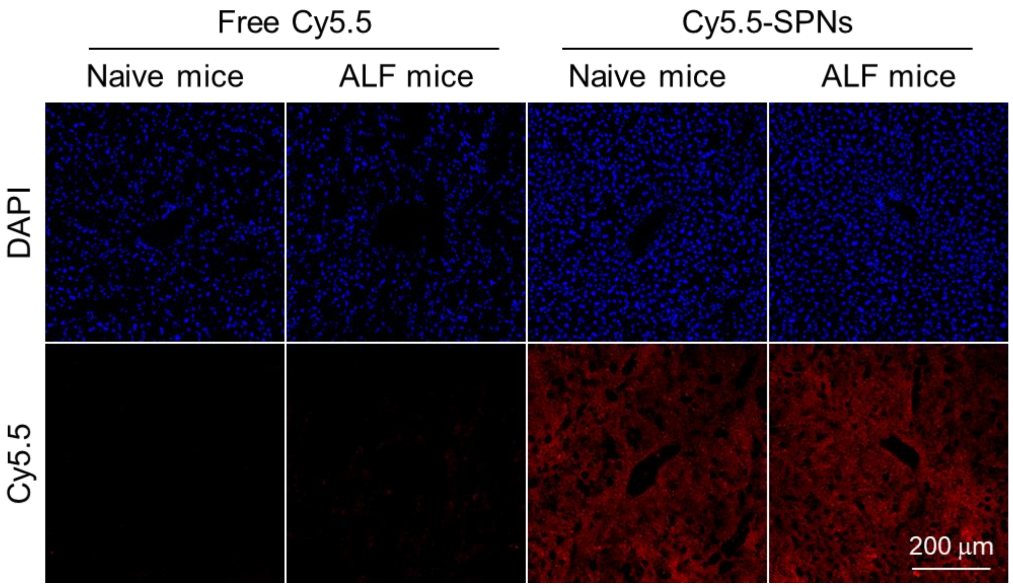

Figure S10. Channel images showing representative confocal microscopy images of liver tissues after 9 h of intravenous injection of free Cy5.5 or Cy5.5-SPNs.

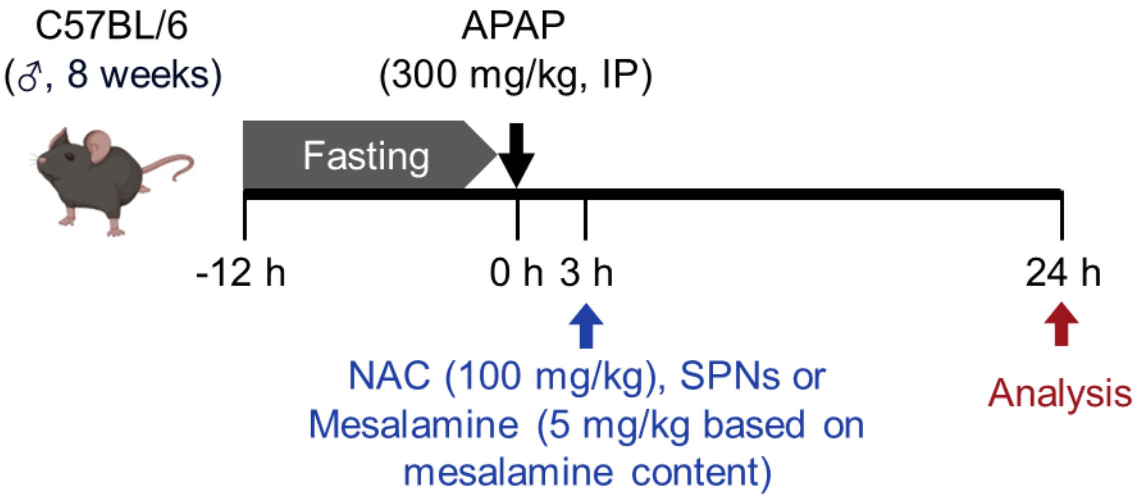

Figure S11. Treatment schedule to assess the therapeutic efficacy of SPNs in ALF mice.

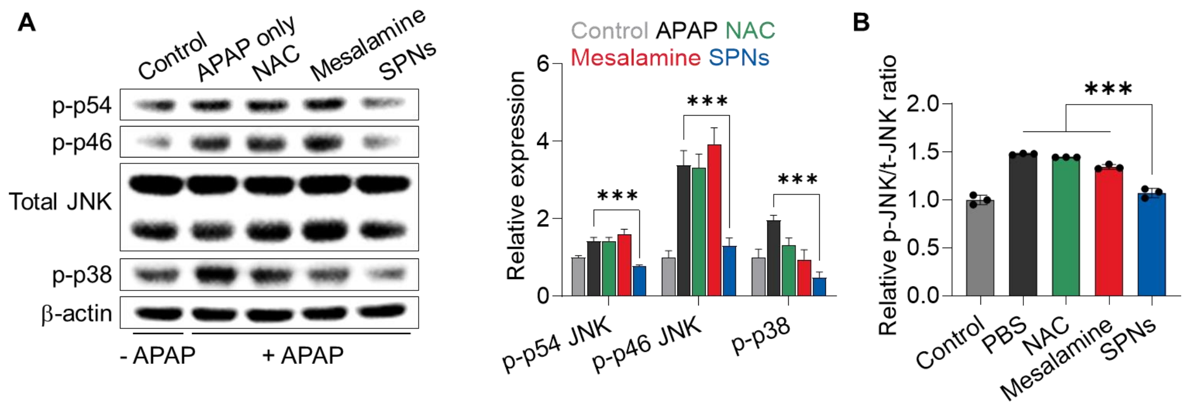

Figure S12. (A) Expression levels of total and phosphorylated JNKs and (B) the relative ratio of phosphorylated JNK to total JNK levels in the liver tissues of ALF mice at 24 h post-treatment with PBS, NAC, mesalamine or SPNs.

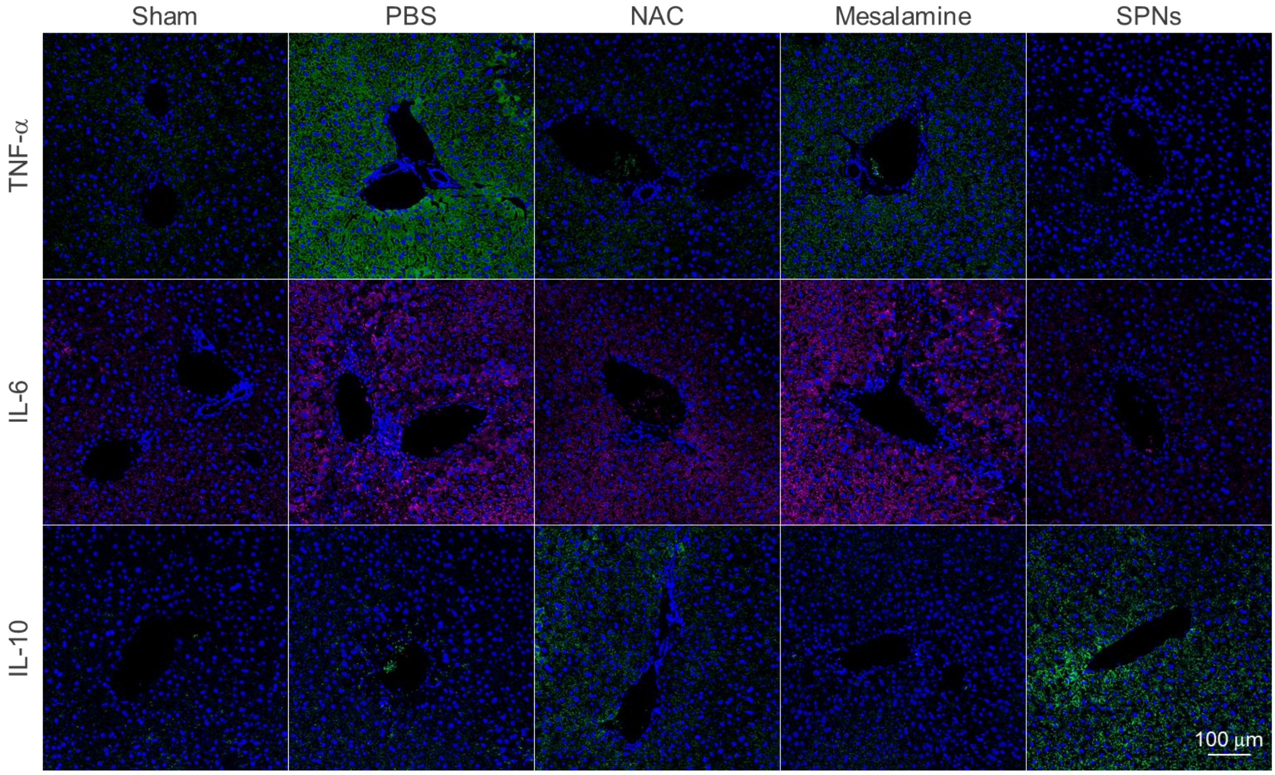

Figure S13. Histological analysis showing pro- and anti-inflammatory cytokines in liver tissues of ALF mice treated with NAC, mesalamine and SPNs.

	AST (IU/L)	ALT (IU/L)	BUN (mg/dL)	Creatinine (mg/dL)	LDH (IU/L)	CK (IU/L)
Control	38.67 ± 3.95	26.53 ± 3.80	24.64 ± 4.11	0.32 ± 0.01	130.67 ± 6.06	128.93 ± 9.91
Mesalamine*1	49.2 ± 1.56	35.60 ± 3.14	29.93 ± 2.00	0.50 ± 0.01	187.13 ± 19.02	158.93 ± 4.80
Mesalamine*3	49.80 ± 1.97	34.87 ± 3.14	27.65 ± 1.38	0.50 ± 0.01	197.47 ± 13.60	213.13 ± 14.25
SPNs*1	39.47 ± 1.97	29.2 ± 0.80	27.03 ± 1.06	0.27 ± 0.09	141.87 ± 20.46	131.20 ± 18.61
SPNs*3	37.73 ± 0.46	27.47 ± 0.23	23.29 ± 1.02	0.27 ± 0.09	131.20 ± 6.44	125.73 ± 7.57

Figure S14. Detail information on hematological parameters.

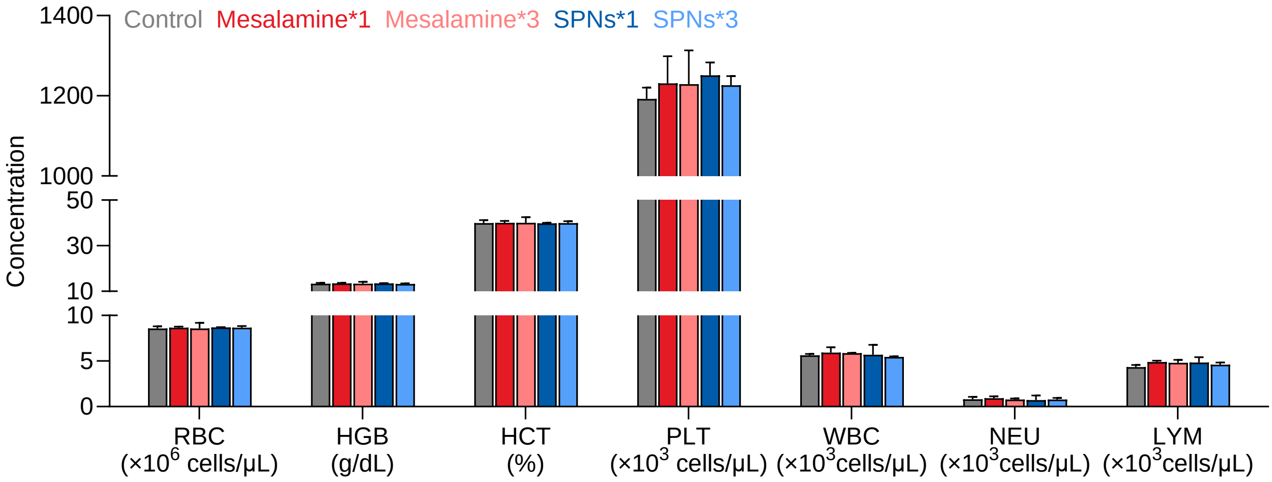

Figure S15. Levels of routine blood components of mice treated with single and multiple doses of mesalamine or SPNs.

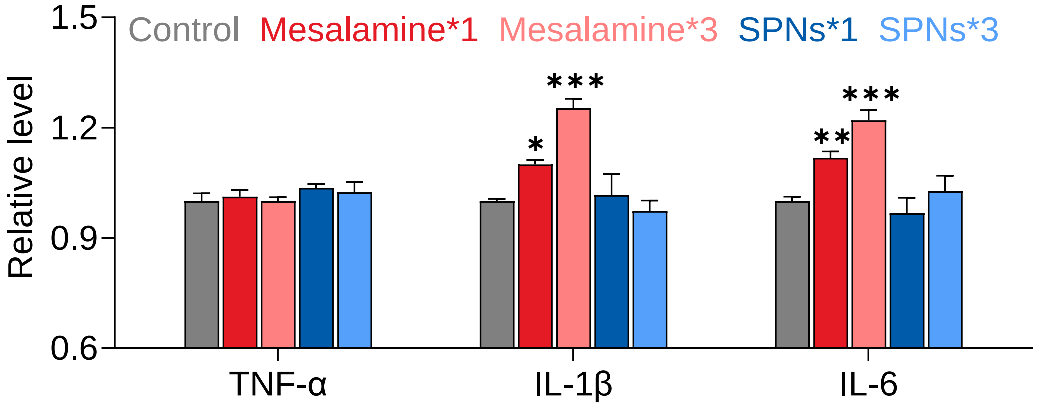

Figure S16. Levels of inflammatory cytokines in the kidney tissues of mice treated with single and multiple doses of mesalamine or SPNs.

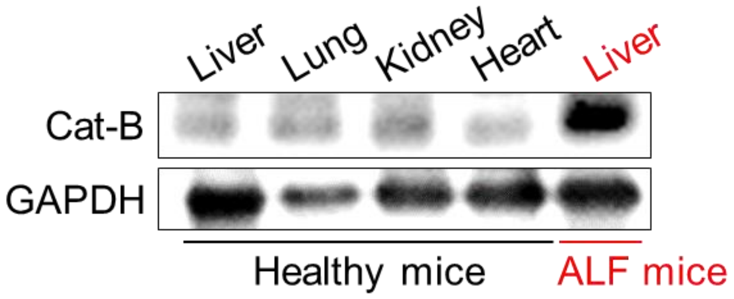

Figure S17. Expression levels of cathepsin B (Cat-B) in healthy organs and in liver tissues exposed to APAP, collected from naive and ALF mice, respectively.

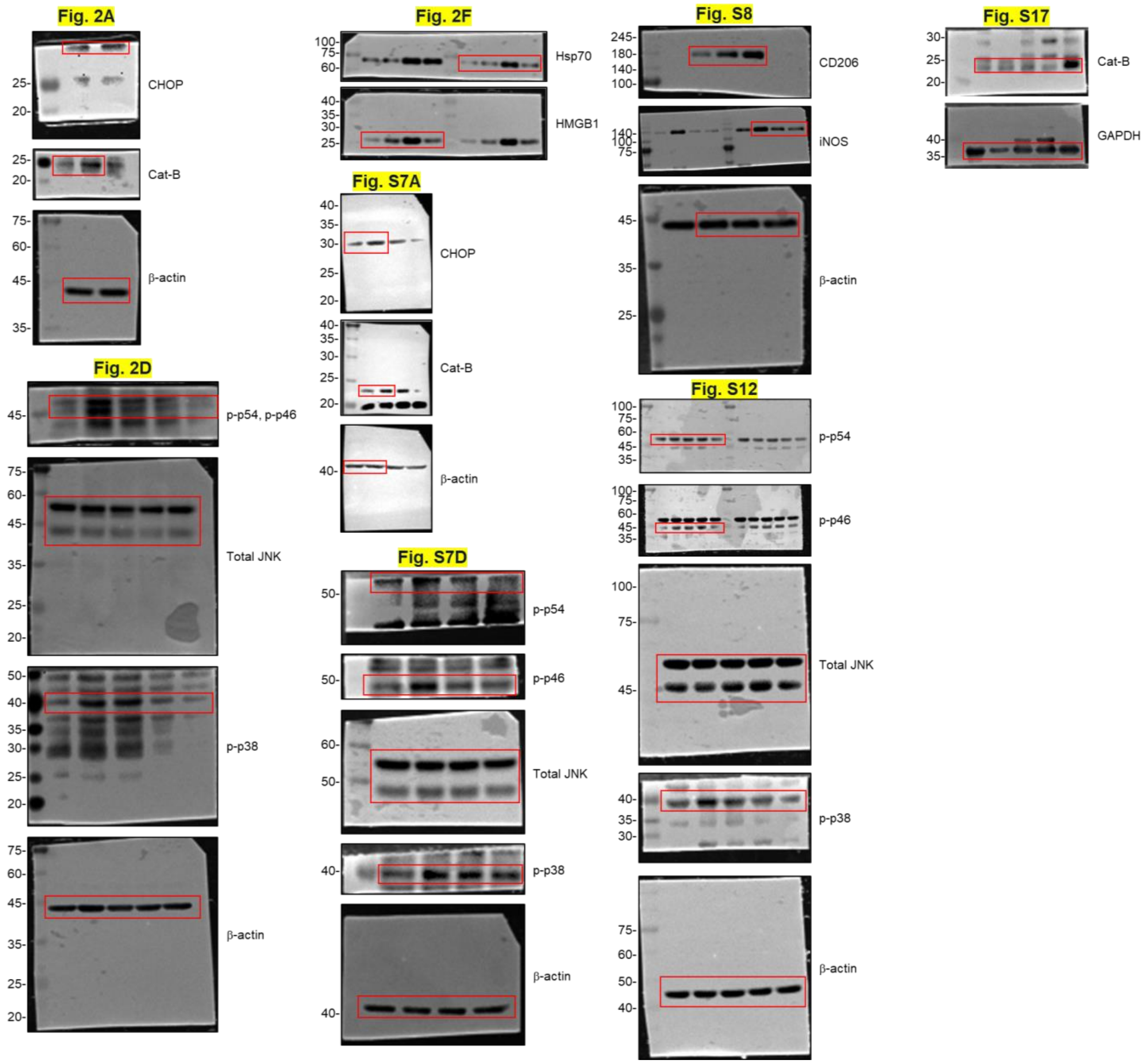

Figure S18. Uncropped original images of Western blot results.
